# Supplementary material for: Jak-Stat pathway induces Drosophila follicle elongation by a gradient of apical contractility
Source: eLife. 2018 Feb 8;7:e32943. doi: 10.7554/eLife.32943 (PMC5805408; doi:10.7554/eLife.32943)
Supplement: Supplementary file 1. — The Baz-Cherry fusion protein was produced by cloning mCherry in frame at the C-terminus of the Par-6 coding sequence in the pUASP vector. [file elife-32943-supp1.docx]

**Supplementary file 1: stock list and source**

| Stock | Genotype | Source / Reference |
| --- | --- | --- |
| *Fat2* mutant | *fat2^58D^ / TM6B* | Dahmann lab / (Viktorinová et al., 2009) |
| *Pak* mutants (2 alleles) (null) | *Pak^11^ /TM3, Sb* and *Pak^6^/TM3 ,Sb, Ser* | BDSC / (Hing et al., 1999) |
| *Df(Pvr)* | *w^1118^; Df(2L)BSC227/CyO* | BDSC |
| *mys* mutant (null) | *FRT101,* *mys^XG43^ / FM7h ; βv1/CyO* | Brown Lab / (Bunch et al., 1992) |
| Tj :GAL4 | *y ,w, ; P(GawB)NP1624* | DGRC Kyoto |
| Upd:GAL4 | *P{upd1-GAL4.U}* | Pret Lab |
| Fru:GAL4 | [*TI^GAL4.P1.D^*](http://flybase.org/reports/FBti0168666.html){GAL4} | Pret Lab / (Boquet et al., 2000) |
| Stat RNAi | *P{GD4492}v43866* | VDRC |
| Upd RNAi | *P{TRiP.JF03149}attP2* | BDSC |
| UAS :HopTum | *P{UAS-hop.Tum} / CyO* | Harisson lab / (Harrison et al., 1995) |
| UAS :Upd | *P{UAS-Upd1}PK9* | Harisson lab |
| STAT10X:GFP | *P{10XStat92E-GFP}* | Crozatier M / (Bach et al., 2007) |
| sqhRNAi | *P{TRiP.HMS00830}attP2* | BDSC |
| BazTrap | [*P01941*](http://flybase.org/reports/FBst0051572.html){PTT-GC} | BDSC / (Buszczak et al., 2007) |
| SqhGFP | *sqh^Ax 3^;; P{sqh-GFP.RLC}* | Karess lab / (Royou et al., 2004) |
| SqhCherry | *sqh^Ax 3^;; P{sqh-mCherry.M}3* | Wieschaus Lab / (Martin et al., 2009) |
| UAS :Baz-Cherry | *P{UASp-Baz Cherry}III* | This study* |
| *rok* mutant (null) | *FRT9-2, rok^2^ / FM0* | Karess lab / (Winter et al., 2001) |
| *sqh* mutant (null) | *FRT101, Sqh^AX3^ / FM7h* | Karess lab / (Jordan and Karess, 1997) |
| *Stat* mutant (null) | *FRT82B, Stat92E^397^ / TM6B* | Montell lab / (Silver and Montell, 2001) |
| DE-Cad-GFP | *TI (Tiainen et al., 1999)shg^GFP^* | B Aigouy / (Huang et al., 2009) |
| Ubi:H2A-mRFP; Ubi:spd-2-GFP | *P{Ubi:H2A-mRFP}; P{Ubi:spd-2-GFP}* | Basto lab / (Dix and Raff, 2007) |
