# Supplementary material for: Jak-Stat pathway induces Drosophila follicle elongation by a gradient of apical contractility
Source: eLife. 2018 Feb 8;7:e32943. doi: 10.7554/eLife.32943 (PMC5805408; doi:10.7554/eLife.32943)
Supplement: Supplementary file 2. — HS: 1 hr heat-shock at 37°C, when not specified flies were kept at 25°C. [file elife-32943-supp2.docx]

**Supplementary file 2: detailed genotypes and specific conditions**

| **Figure** | | **Genotype** | | **Conditions** |
| --- | --- | --- | --- | --- |
| **Figure 1** | a | WT | |  |
|  | b | WT | |  |
|  | c, d | *fat2^58D^/fat2^58D^* | |  |
|  | e | *pak^6^/pak^11^* | |  |
|  | f,g | *Df(Pvr)/+; Pak^6^/Pak^11^* | |  |
|  | h, i ,j | *FRT101, e22c:GAL4, UAS:flp/FRT101, mys^XG43^; βv^1^/+* | |  |
|  |  |  | |  |
| **Figure 2** | a | WT | |  |
|  | b, h | 10X Stat:GFP | |  |
|  | c, g | Upd:GAL4/+; RNAi upd1^JF03149^ | | Cross at 25°C, 7 days at 30°C |
|  | d, g | Tub:GAL80^TS^/RNAi Stat92E ^T1B510^; Fru:GAL4/+ | | Cross at 18°C, 3 days at 30°C |
|  | e | *y,w,HS:flp122/+ ; tub:FRT-stop-FRT-GAL4, UAS:GFP/+; [UAS:upd]^pk9^/+* | | 1HS, 3 days after HS |
|  | f,g | Tj:GAL4/UAS:Hop^tum^ | | Cross at 25°C, 3 days at 30°C |
|  |  |  | |  |
| **Figure 3** | a | WT | |  |
|  | a', b | Tj:GAL4/+; UAS: RNAi Sqh^HMS00830^/+ | | Cross at 25°C, 6 days at 30°C |
|  | c-f, j | Baz-GFP, *sqh^Ax3^;* Sqh-mCherry | |  |
|  | g, h | Baz-GFP | |  |
|  |  |  | |  |
| **Figure 4** | a-g | Baz-GFP | |  |
|  | h, i | Baz-GFP | | 5 days at 30°C |
|  |  | Baz-GFP/+; Tj:GAL4/+; RNAi upd1^JF03149^ | | Cross at 25°C, 5 days at 30°C |
|  | j | *y,w,HSflp122/+;* *tub:FRT-stop-FRT-GAL4, UAS:GFP/*+*;* UAS: Baz-mCherry | | 1HS, 3 days after HS at 25°C |
|  |  | *y,w,HSflp122/+;* *tub:FRT-stop-FRT-GAL4, UAS:GFP/*UAS:Hop^tum^*;* UAS: Baz-mCherry | | 1HS, 3 days after HS at 25° |
|  |  |  | |  |
| **Figure 5** | a | *y,w,HS:flp122/+;;FRT82B, Ubi:RFP^nls^/FRT82B, Sta92E^397^* | | 1HS, 5 days after HS |
|  | b | *y,w,HSflp122/+;* *tub:FRT-stop-FRT-GAL4, UAS:GFP/*UAS:Hop^tum^ | | 1HS, 3 days after HS |
|  | c,d ,e,f | WT | |  |
|  | f,h,i | *y,w, FRT101 Ubi:GFP/ FRT101 Sqh^AX3^; hsflp/+* | | 1HS, 5 days after HS |
|  | f,j | *y,w, FRT9-2 Ubi:GFP/ FRT9-2 Rok^2^; hsflp/+* | | 1HS, 5 days after HS |
|  |  |  | |  |
| **Figure 6** | a-e | WT | |  |
|  | g,i | Baz-GFP | |  |
|  | i | Baz-GFP/+; Tj:GAL4/+; RNAi upd1^JF03149^ / + | | Cross at 25°C, 5 days at 30°C |
|  | j | *y,w,HS:flp122/+;;FRT82B, Ubi:RFP^nls^/FRT82B, Stat92E^397^* | | 1HS, 10 days after HS |
|  |  |  | |  |
| **Figure 1S1** | a, b | *Fat2^58D^/fat2^58D^* | |  |
|  | c,d | *Df(Pvr)/+; Pak^6^/Pak^11^* | |  |
|  | e,f | *FRT101, e22c:GAL4, UAS:flp/FRT101, mys^XG43^; βv^1^/+* | |  |
|  |  | |  |  |
| **Figure 2S1** | a,d | Tj:GAL4, Tub:GAL80^TS^ / + ; 10xSTAT-GFP /+ | | 2 days at 30°C |
|  | b,d | Tj:GAL4 /+; 10xSTAT-GFP/RNAi upd1^JF03149^ | | Cross at 25°C, 2 days at 30°C |
|  | c,d | Tj:GAL4, Tub:GAL80^TS^ /RNAi Stat92E ^T1B510^; 10xSTAT-GFP/ + | | Cross at 18°C, 2 days at 30°C |
|  | e | WT | |  |
|  |  | Tj:GAL4/+; RNAi upd1^JF03149^ / + | | Cross at 25°C, 5 days at 30°C |
|  |  | Tub:GAL80^TS^/RNAi Stat92E ^T1B510^; Fru:GAL4/+ | | Cross at 18°C, 3 days at 30°C |
|  |  | Tj:GAL4/UAS:Hop^tum^ | | Cross at 25°C, 3 days at 30°C |
|  |  |  | |  |
| **Figure 3S1** | a | WT | |  |
|  | b, c | Tj:GAL4/+; UAS: RNAi Sqh^HMS00830^/+ | | Cross at 25°C, 6 days at 30°C |
|  |  |  | |  |
| **Figure 4S1** | a,b | Baz-GFP | |  |
|  |  |  | |  |
| **Figure 6S1** | a, b, d | DE-Cad-GFP | |  |
|  | c | Ubi:H2A-mRFP; Ubi:spd-2-GFP | |  |
|  | e | Baz-GFP | |  |
