# Supplementary material for: Jak-Stat pathway induces Drosophila follicle elongation by a gradient of apical contractility
Source: eLife. 2018 Feb 8;7:e32943. doi: 10.7554/eLife.32943 (PMC5805408; doi:10.7554/eLife.32943)
Supplement: Supplementary file 3. — n corresponds to the number of analyzed follicles, with usually more than 10 segmented cells per follicle. [file elife-32943-supp3.docx]

**Supplementary file 3 : detailed sample size**

| **Figure** | |  | | | | |  | | |  |  |  |  |  |  |  |  |  |  |  |  |  |  |  |  |  |  |  |  |  |  |  |  |  |  |  |  |  |  |  |  |  |  |  |  |  |  |
| --- | --- | --- | --- | --- | --- | --- | --- | --- | --- | --- | --- | --- | --- | --- | --- | --- | --- | --- | --- | --- | --- | --- | --- | --- | --- | --- | --- | --- | --- | --- | --- | --- | --- | --- | --- | --- | --- | --- | --- | --- | --- | --- | --- | --- | --- | --- | --- |
| **1d** | | **Stage** | | | | | **WT** | | | | | ***Fat2*** | |  | | | | | |  | | | |  |  |  |  |  |  |  |  |  |  |  |  |  |  |  |  |  |  |  |  |  |  |  |  |
|  | | 3 | | | | | 43 | | | | | **20** | |  | | | | | |  | | | |  |  |  |  |  |  |  |  |  |  |  |  |  |  |  |  |  |  |  |  |  |  |  |  |
|  | | 4 | | | | | 39 | | | | | 26 | |  | | | | | |  | | | |  |  |  |  |  |  |  |  |  |  |  |  |  |  |  |  |  |  |  |  |  |  |  |  |
|  | | 5 | | | | | 20 | | | | | 18 | |  | | | | | |  | | | |  |  |  |  |  |  |  |  |  |  |  |  |  |  |  |  |  |  |  |  |  |  |  |  |
|  | | 6 | | | | | 20 | | | | | 11 | |  | | | | | |  | | | |  |  |  |  |  |  |  |  |  |  |  |  |  |  |  |  |  |  |  |  |  |  |  |  |
|  | | 7 | | | | | 21 | | | | | 25 | |  | | | | | |  | | | |  |  |  |  |  |  |  |  |  |  |  |  |  |  |  |  |  |  |  |  |  |  |  |  |
|  | | 8 | | | | | 26 | | | | | 38 | |  | | | | | |  | | | |  |  |  |  |  |  |  |  |  |  |  |  |  |  |  |  |  |  |  |  |  |  |  |  |
|  | | 9 | | | | | 40 | | | | | 10 | |  | | | | | |  | | | |  |  |  |  |  |  |  |  |  |  |  |  |  |  |  |  |  |  |  |  |  |  |  |  |
|  | | 10 | | | | | 7 | | | | | 10 | |  | | | | | |  | | | |  |  |  |  |  |  |  |  |  |  |  |  |  |  |  |  |  |  |  |  |  |  |  |  |
|  | | 10B | | | | | 13 | | | | | 10 | |  | | | | | |  | | | |  |  |  |  |  |  |  |  |  |  |  |  |  |  |  |  |  |  |  |  |  |  |  |  |
|  | | 11 | | | | | 18 | | | | | 8 | |  | | | | | |  | | | |  |  |  |  |  |  |  |  |  |  |  |  |  |  |  |  |  |  |  |  |  |  |  |  |
|  | | 12 | | | | | 6 | | | | | 7 | |  | | | | | |  | | | |  |  |  |  |  |  |  |  |  |  |  |  |  |  |  |  |  |  |  |  |  |  |  |  |
|  | | 13 | | | | | 13 | | | | | 7 | |  | | | | | |  | | | |  |  |  |  |  |  |  |  |  |  |  |  |  |  |  |  |  |  |  |  |  |  |  |  |
|  | | 14 | | | | | 48 | | | | | 15 | |  | | | | | |  | | | |  |  |  |  |  |  |  |  |  |  |  |  |  |  |  |  |  |  |  |  |  |  |  |  |
| **1j, 1S1** | |  | | | | | **WT-like polar cells** | | | | | **Mislocalized polar cells** | | | | | | |  | | | | | | | | | | | | | |  | | | |  | | | | | | |  | | | |
|  | | ***Mys^XG43^* clones** | | | | | 34 | | | | | 31 | | | | | | |  | | | | | | | | | | | | | |  | | | |  | | | | | | |  | | | |
|  | |  | | | | |  | | | | |  | | | | | | |  | | | | | | | | | | | | | |  | | | |  | | | | | | |  | | | |
| **1g, 1S1** | |  | | | | | **WT-like polar cells** | | | | | **Mislocalized polar cells** | | | | | | |  | | | | | | | | | | | | | |  | | | |  | | | | | | |  | | | |
|  | | ***Df(Pvr)*;*pak^6/11^*** | | | | | 13 | | | | | 23 | | | | | | |  | | | | | | | | | | | | | |  | | | |  | | | | | | |  | | | |
|  | |  | | | | |  | | | | |  | | | | | | |  | | | | | | | | | | | | | |  | | | |  | | | | | | |  | | | |
|  | |  | |  | | | | | | | |  | | | | | | |  | | |  | | | | | | |  | | | | | | | | |  |  |  |  |  |  |  |  |  |  |
| **2g** | | **Each control**  **(U or G)** | | **Upd:GAL4;**  **UpdRNAi** | | | | | | | | **Tj:GAL4;**  **Hop^tum^** | | | | | | | **Fru:GAL4;**  **Stat92E RNAi** | | | | | | | |  | | | | | |  |  |  |  |  |  |  |  |  |  |  |  |  |  |  |
|  | | 30 | | 41 | | | | | | | | 51 | | | | | | | 97 | | | | | | | |  | | | | | |  |  |  |  |  |  |  |  |  |  |  |  |  |  |  |
|  | |  | | | | |  | | | | |  | | | | | | |  | | | |  | | | | | | | |  | | | | | | | | |  |  |  |  |  |  |  |  |
| **2j** | | **Stage 3** | | | | | **Stage 5** | | | | | **Stage 7** | | | | | | |  | | | |  | | | | | | | |  | | | | | | | | |  |  |  |  |  |  |  |  |
|  | | 5 | | | | | 5 | | | | | 6 | | | | | | |  | | | |  | | | | | | | |  | | | | | | | | |  |  |  |  |  |  |  |  |
|  | |  | | | | |  | | | | |  | | | | | | |  | | | |  | | | | | | | |  | | | | | | | | |  |  |  |  |  |  |  |  |
| **3b** | | **Tj:GAL4** | | **Sqh RNAi** | | | | | | | | **Tj:GAL4;Sqh RNAi** | | | | | | |  | | | | | | | |  | | | | | |  |  |  |  |  |  |  |  |  |  |  |  |  |  |  |
|  | | 65 | | 30 | | | | | | | | 46 | | | | | | |  | | | | | | | |  | | | | | |  |  |  |  |  |  |  |  |  |  |  |  |  |  |  |
|  | |  | | | | |  | | | | |  | | | | | | |  | | | | | | | | | |  | | | | | | |  | | | | | | |  | | | |  |
| **3f** | | 6 follicles | | | | | 86 cells | | | | |  | | | | | | |  | | | | | | | | | |  | | | | | | |  | | | | | | |  | | | |  |
|  | |  | | | | |  | | | | |  | | | | | | |  | | | | | | | | | |  | | | | | | |  | | | | | | |  | | | |  |
| **3g** | | **Control** | | | | | **Y-27632** | | | | |  | | | | | | |  | | | | | | | | | |  | | | | | | |  | | | | | | |  | | | |  |
|  | | 13 | | | | | 7 | | | | |  | | | | | | |  | | | | | | | | | |  | | | | | | |  | | | | | | |  | | | |  |
|  | |  | | | | |  | | | | |  | | | | | | |  | | | | | | | | | |  | | | | | | |  | | | | | | |  | | | |  |
| **3h** | | **Stage 3-4 ML** | | | | | **Stage 7-8 ML** | | | | | **Stage 7-8 pole** | | | | | | |  | | | | | | | | | |  | | | | | | |  | | | | | | |  | | | |  |
|  | | 14 | | | | | 16 | | | | | 15 | | | | | | |  | | | | | | | | | |  | | | | | | |  | | | | | | |  | | | |  |
|  | |  | | | | |  | | | | |  | | | | | | |  | | | | | | | | | |  | | | | | | |  | | | | | | |  | | | |  |
| **3i** | | **Baz-GFP** | | | | | **Sqh-mCherry** | | | | |  | | | | | | |  | | | | | | | | | |  | | | | | | |  | | | | | | |  | | | |  |
|  | | 44 | | | | | 41 | | | | |  | | | | | | |  | | | | | | | | | |  | | | | | | |  | | | | | | |  | | | |  |
|  | |  | | | | |  | | | | |  | | | | | | |  | | | | | | | | | |  | | | | | | |  | | | | | | |  | | | |  |
| **4f** | | **St3-4 ML** | | | | | **St7-8 ML** | | | | | **St3-4 pole** | | | | | | | **St 7-8 pole** | | | | | | | | | |  | | | | | | |  | | | | | | |  | | | |  |
|  | | 9 | | | | | 10 | | | | | 11 | | | | | | | 16 | | | | | | | | | |  | | | | | | |  | | | | | | |  | | | |  |
|  | |  | | | | |  | | | | |  | | | | | | |  | | | | | | | | | |  | | | | | | |  | | | | | | |  | | | |  |
| **4h** | | **WT St3-4 ML** | | | | | **Upd RNAi St3-4 ML** | | | | |  | | | | | | |  | | | | | | | |  | | |  | | | | | | | | |  |  |  |  |  |  |  |  |  |
|  | | 16 | | | | | 9 | | | | |  | | | | | | |  | | | | | | | |  | | |  | | | | | | | | |  |  |  |  |  |  |  |  |  |
| **4i** | | **WT St 7-8 pole** | | | | | **Upd RNAi St 7-8 pole** | | | | |  | | | | | | |  | | | | | | | |  | | |  | | | | | | | | |  |  |  |  |  |  |  |  |  |
|  | | 16 | | | | | 9 | | | | |  | | | | | | |  | | | | | | | |  | | |  | | | | | | | | |  |  |  |  |  |  |  |  |  |
| **4j** | | **Control** | | | | | **Flipout Hop^tum^** | | | | |  | | | | | | |  | | | | | | | | | | | | | |  | | | |  | | | | | | |  | | | |
|  | | 8 | | | | | 5 | | | | |  | | | | | | |  | | | | | | | | | | | | | |  | | | |  | | | | | | |  | | | |
|  | |  | | | | |  | | | | |  | | | | | | |  | | | | | | | | | | | | | |  | | | |  | | | | | | |  | | | |
| **5d** | | 2 measures/follicle | | | | | 5 follicles | | | | |  | | | | | | |  | | | | | | | | | | | | | |  | | | |  | | | | | | |  | | | |
|  | |  | | |  | | | | | | |  | | | | | | |  | |  | | | | | | | | | | |  | | | | | | |  |  |  |  |  |  |  |  |  |
| **5f** | | **WT** | | | ***Sqh* anterior clone** | | | | | | | ***Rok* anterior clone** | | | | | | |  | |  | | | | | | | | | | |  | | | | | | |  |  |  |  |  |  |  |  |  |
|  | | 16 | | | 10 | | | | | | | 11 | | | | | | |  | |  | | | | | | | | | | |  | | | | | | |  |  |  |  |  |  |  |  |  |
|  | |  | | | | |  | | | | |  | | | | | | |  | | | | | | | | | | | | | |  | | | |  | | | | | | |  | | | |
| **6d** | | **Stage 6** | | | | | **Stage 7** | | | | | **Stage 8** | | | | | | |  | | | | | | | | | | | | | |  | | | |  | | | | | | |  | | | |
|  | | 44 | | | | | 97 | | | | | 130 | | | | | | |  | | | | | | | | | | | | | |  | | | |  | | | | | | |  | | | |
|  | |  | | | | |  | | | | |  | | | | | | |  | | | | | | | | | | | | | |  | | | |  | | | | | | |  | | | |
| **6e** | | 271 | | | | |  | | | | |  | | | | | | |  | | | | | | | | | | | | | |  | | | |  | | | | | | |  | | | |
|  | |  | | | | |  | | | | |  | | | | | | |  | | | | | | | | | | | | | |  | | | |  | | | | | | |  | | | |
|  | |  | | | | |  | | | | |  | | | | | | |  | | | | | | | | | | | | | |  | | | |  | | | | | | |  | | | |
| **6g** | | 10 follicles | | | | | 1487 cells | | | | |  | | | | | | |  | | | | | | | | | | | | | |  | | | |  | | | | | | |  | | | |
|  | |  | | | | |  | | | | |  | | | | | | |  | | | | | | | | | | | | | |  | | | |  | | | | | | |  | | | |
| **6h** | | **St3-4 ML** | | | | | **St3-4 pole** | | | | | **St7-8 ML** | | | | | | | **St 7-8 pole** | | | | | | | **St 7-8 pole RNAi Upd** | | | | | | | | |  | | | | | | |  | | |  |  |  |
|  | | 9 | | | | | 11 | | | | | 9 | | | | | | | 10 | | | | | | | 10 | | | | | | | | |  | | | | | | |  | | |  |  |  |
|  | |  | | | | |  | | | | |  | | | | | | |  | | | | | | | | | | | | | |  | | | |  | | | | | | |  | | | |
| **6i** | | **For all conditions** | | | | |  |  | | | |  | | | | | | |  |  |  |  |  |  |  |  |  |  |  |  |  |  |  |  |  |  |  |  |  |  |  |  |  |  |  |  |  |
|  | | 2 measures/follicle | | | | |  |  | | | |  | | | | | | |  |  |  |  |  |  |  |  |  |  |  |  |  |  |  |  |  |  |  |  |  |  |  |  |  |  |  |  |  |
|  | | 6 follicles | | | | |  |  | | | |  | | | | | | |  |  |  |  |  |  |  |  |  |  |  |  |  |  |  |  |  |  |  |  |  |  |  |  |  |  |  |  |  |
|  | |  | | | | |  | | | | |  | | | | | | |  | | | | | | | | | | | | | |  | | | |  | | | | | | |  | | | |
| **6k** | | 10 stage 3-5 follicles | | | | |  | | | | |  | | | | | | |  | | | | | | | | | | | | | |  | | | |  | | | | | | |  | | | |
|  | |  | | | | |  | | | | |  | | | | | | |  | | | | | | | | | | | | | |  | | | |  | | | | | | |  | | | |
| **6m** | | 12 stage 3-5 follicles | | | | | 3 measure/follicle/ genotype | | | | |  | | | | | | |  | | | | | | | | | | | | | |  | | | |  | | | | | | |  | | | |
|  | |  | | | | |  | | | | |  | | | | | | |  | | | | | | | | | | | | | |  | | | |  | | | | | | |  | | | |
| **2S1d** | | **WT** | | | | | **Upd RNAi** | | | | | **Stat RNAi** | | | | | | |  | | | | | | | | | | | | | |  | | | |  | | | | | | |  | | | |
|  | | 14 | | | | | 8 | | | | | 9 | | | | | | |  | | | | | | | | | | | | | |  | | | |  | | | | | | |  | | | |
|  | |  | | | |  | | | | |  | | | |  | | | | | | | | | | | | | | | | | | |  |  | |  | | | | | | | | |  |  |
| **2S1e** | **Stage** | | **WT** | | | | | | **Upd RNAi** | | | | | | **Hop^tum^** | | | **Stat RNAi** | | | | | | | | | |  | | | | | | | | | | | | |  |  |  |  |  |  |  |
|  | 3 | | 43 | | | | | | 18 | | | | | | 7 | | | 29 | | | | | | | | | |  | | | | | | | | | | | | |  |  |  |  |  |  |  |
|  | 4 | | 39 | | | | | | 15 | | | | | | 11 | | | 15 | | | | | | | | | |  | | | | | | | | | | | | |  |  |  |  |  |  |  |
|  | 5 | | 20 | | | | | | 20 | | | | | | 12 | | | 20 | | | | | | | | | |  | | | | | | | | | | | | |  |  |  |  |  |  |  |
|  | 6 | | 20 | | | | | | 10 | | | | | | 5 | | | 18 | | | | | | | | | |  | | | | | | | | | | | | |  |  |  |  |  |  |  |
|  | 7 | | 21 | | | | | | 12 | | | | | | 16 | | | 15 | | | | | | | | | |  | | | | | | | | | | | | |  |  |  |  |  |  |  |
|  | 8 | | 26 | | | | | | 11 | | | | | | 7 | | | 7 | | | | | | | | | |  | | | | | | | | | | | | |  |  |  |  |  |  |  |
|  | 9 | | 40 | | | | | | 11 | | | | | | 10 | | | 8 | | | | | | | | | |  | | | | | | | | | | | | |  |  |  |  |  |  |  |
|  | |  | | | | |  | | | | |  | | | | | | |  | | | |  | | | | | | | |  | | | | | | | | |  |  |  |  |  |  |  |  |
| **3S1c** | | **Stage** | | | | | **WT** | | | | | **Sqh RNAi** | | | | | | |  | | | |  | | | | | | | |  | | | | | | | | |  |  |  |  |  |  |  |  |
|  | | 3 | | | | | 43 | | | | | 8 | | | | | | |  | | | |  | | | | | | | |  | | | | | | | | |  |  |  |  |  |  |  |  |
|  | | 4 | | | | | 39 | | | | | 14 | | | | | | |  | | | |  | | | | | | | |  | | | | | | | | |  |  |  |  |  |  |  |  |
|  | | 5 | | | | | 20 | | | | | 9 | | | | | | |  | | | |  | | | | | | | |  | | | | | | | | |  |  |  |  |  |  |  |  |
|  | | 6 | | | | | 20 | | | | | 9 | | | | | | |  | | | | | | | | | | | | | |  | | | |  | | | | | | |  | | | |
|  | | 7 | | | | | 21 | | | | | 6 | | | | | | |  | | | | | | | | | | | | | |  | | | |  | | | | | | |  | | | |
|  | | 8 | | | | | 26 | | | | | 14 | | | | | | |  | | | | | | | | | | | | | |  | | | |  | | | | | | |  | | | |
|  | | 9 | | | | | 40 | | | | | 9 | | | | | | |  | | | | | | | | | | | | | |  | | | |  | | | | | | |  | | | |
|  |  | |  | | | | | | | |  | | | |  | | | | | | | | | | | | | | | | | | |  |  | |  | | | | | | | | |  |  |
| **4S1a** | | 35 follicles | | | | |  | | | | | |  | | |  |  | | | | | | | |  |  |  |  |  |  |  |  |  |  |  |  |  |  |  |  |  |  |  |  |  |  |  |
|  | |  | | | | |  | | | | |  | | | | | | |  | | | | | | | | | | | | | |  | | | |  | | | | | | |  | | | |
| **4S1b** | | 5 follicles | | | | | 441 cells | | | | |  | | | | | | |  | | | | | | | | | | | | | |  | | | |  | | | | | | |  | | | |
|  | |  | | | | |  | | | | |  | | | | | | |  | | | |  | | | | | | | |  | | | | | | | | |  |  |  |  |  |  |  |  |
| **6S1a,b** | | **Stage** | | | | | **WT** | | | | |  | | | | | | |  | | | |  | | | | | | | |  | | | | | | | | |  |  |  |  |  |  |  |  |
|  | | 3 | | | | | 31 | | | | |  | | | | | | |  | | | |  | | | | | | | |  | | | | | | | | |  |  |  |  |  |  |  |  |
|  | | 4 | | | | | 21 | | | | |  | | | | | | |  | | | |  | | | | | | | |  | | | | | | | | |  |  |  |  |  |  |  |  |
|  | | 5 | | | | | 19 | | | | |  | | | | | | |  | | | |  | | | | | | | |  | | | | | | | | |  |  |  |  |  |  |  |  |
|  | | 6 | | | | | 23 | | | | |  | | | | | | |  | | | | | | | | | | | | | |  | | | |  | | | | | | |  | | | |
|  | | 7 | | | | | 25 | | | | |  | | | | | | |  | | | | | | | | | | | | | |  | | | |  | | | | | | |  | | | |
|  | | 8 | | | | | 25 | | | | |  | | | | | | |  | | | | | | | | | | | | | |  | | | |  | | | | | | |  | | | |
|  | |  | | | | |  | | | | |  | | | | | | |  | | | | | | | | | | | | | |  | | | |  | | | | | | |  | | | |
| **6S1d** | | **Stage 3-5** | | | | | 202 divisions | | | | |  | | | | | | |  | | | | | | | | | | | | | |  | | | |  | | | | | | |  | | | |
